# Curcumin analogs (B2BrBC and C66) supplementation attenuates airway hyperreactivity and promote airway relaxation in neonatal rats exposed to hyperoxia

**DOI:** 10.14814/phy2.14555

**Published:** 2020-08-18

**Authors:** Mimoza Stamenkovska, Qendrim Thaçi, Nikola Hadzi‐Petrushev, Marija Angelovski, Jane Bogdanov, Shkëlzen Reçica, Islam Kryeziu, Hristo Gagov, Vadim Mitrokhin, Andre Kamkin, Rudolf Schubert, Mitko Mladenov, Ramadan B. Sopi

**Affiliations:** ^1^ Faculty of Natural Sciences and Mathematics Institute of Biology “Sts, Cyril and Methodius” University Skopje Macedonia; ^2^ Department of Premedical Courses‐Biology Faculty of Medicine University of Prishtina St. Martyrs’ Boulevard n.n. Prishtina Kosovo Serbia; ^3^ Faculty of Natural Sciences and Mathematics Institute of Chemistry “Ss. Cyril and Methodius” University Skopje Macedonia; ^4^ Faculty of Biology Sofia University St. Kliment Ohridski Sofia Bulgaria; ^5^ Department of Fundamental and Applied Physiology Russian National Research Medical University Moscow Russia; ^6^ Physiology Institute of Theoretical Medicine Medical Faculty University of Augsburg Augsburg Germany

**Keywords:** bronchopulmonary dysplasia, catalase, curcuminoids, tracheal smooth muscle

## Abstract

**Background:**

This study was undertaken to test the hypothesis that the newly synthesized curcuminoids B2BrBC and C66 supplementation will overcome hyperoxia‐induced tracheal hyperreactivity and impairment of relaxation of tracheal smooth muscle (TSM).

**Materials and methods:**

Rat pups (P5) were exposed to hyperoxia (>95% O_2_) or normoxia for 7 days. At P12, tracheal cylinders were used to study in vitro contractile responses induced by methacholine (10^−8^–10^−4^M) or relaxation induced by electrical field stimulation (5–60 V) in the presence/absence of B2BrBC or C66, or to study the direct relaxant effects elicited by both analogs.

**Results:**

Hyperoxia significantly increased contraction and decreased relaxation of TSM compared to normoxia controls. Presence of B2BrBC or C66 normalized both contractile and relaxant responses altered by hyperoxia. Both, curcuminoids directly induced dose‐dependent relaxation of preconstricted TSM. Supplementation of hyperoxic animals with B2BrBC or C66, significantly increased catalase activity. Lung TNF‐α was significantly increased in hyperoxia‐exposed animals. Both curcumin analogs attenuated increases in TNF‐α in hyperoxic animals.

**Conclusion:**

We show that B2BrBC and C66 provide protection against adverse contractility and relaxant effect of hyperoxia on TSM, and whole lung inflammation. Both analogs induced direct relaxation of TSM. Through restoration of catalase activity in hyperoxia, we speculate that analogs are protective against hyperoxia‐induced tracheal hyperreactivity by augmenting H_2_O_2_ catabolism. Neonatal hyperoxia induces increased tracheal contractility, attenuates tracheal relaxation, diminishes lung antioxidant capacity, and increases lung inflammation, while monocarbonyl CUR analogs were protective of these adverse effects of hyperoxia. Analogs may be promising new therapies for neonatal hyperoxic airway and lung disease.

## Graphical abstract

This study was undertaken to test the hypothesis that the newly synthesized curcuminoids B2BrBC and C66 supplementation will overcome hyperoxia‐induced airway hyperreactivity and impairment of relaxation of tracheal smooth muscle.

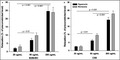

## 1 INTRODUCTION

Chronic lung disease of prematurity, bronchopulmonary dysplasia (BPD), is a result of lung injury complications during the treatment of respiratory failure in premature infants, and is manifested with increased airway reactivity in childhood (Hack et al., 2005; Pelkonen, Hakulinen, & Turpeinen, 1997). In addition to the pre‐ and postnatal inflammatory processes, exposure of immature respiratory tracts to supplemental oxygen plays an important role in the development of BPD (Kinsella, Greenough, & Abman, 2006). Hence, BPD can be induced by hyperoxic exposure throughout the neonatal period of rodents and serves as a model for BPD‐associated airway reactivity. Previously, it was shown by us and other authors that neonatal exposure to a high concentration of oxygen (>95%) is associated with increased contractile responses and decreased relaxant responses of airway smooth muscle (ASM), measured under in vitro and in vivo conditions on extra‐ and intrapulmonary airways (Belik, Jankov, Pan, & Tanswell, 2003; Hershenson et al., 1994; Iben, Dreshaj, Farver, Haxhiu, & Martin, 2000; Mhanna et al., 2004; Sopi et al., 2007, 2008; Vadivel et al., 2010).

Hyperoxic exposure induces reactive oxygen species (ROS) generation in the lungs (Berkelhamer et al., 2013), which is known to be associated with BPD pathophysiology (Nardiello, Mizikova, & Morty, 2017). With respect to the relationship between airway function and persistent oxygen susceptibility, a potential therapeutic approach would be to reduce ROS directly by improving the cellular antioxidant system, in efforts to normalize future airway function. Curcumin (CUR), is a naturally bioactive compound which possesses antioxidant properties (Comhair, Bhathena, Dweik, Kavuru, & Erzurum, 2000). Several studies have reported beneficial biological properties for CUR including antiinflammatory and antioxidant (Chainani‐Wu, 2003), antimicrobial (Han & Yang, 2005), and hepatoprotective effects (Park, Jeon, Ko, Kim, & Sohn, 2000). CUR has been demonstrated to inhibit nitric oxide (NO) production by inducible NO synthase (iNOS) and ROS production in macrophages (Rao, 1997), and to enhance the activity of many antioxidant enzymes such as catalase (CAT), superoxide dismutase (SOD), glutathione peroxidase (GPx) (Reddy & Lokesh, 1994), and heme oxygenase‐1 (HO‐1) (Jeong et al., 2006). Despite these characteristics, it has limited clinical application due to its low bioavailability combined with rapid metabolism and poor chemical stability (Ren & Sowers, 2014). With biochemical engineering, Ren and Sowers, (2014), have found that reducing dicarbonyl with monocarbonyl groups improves both CUR bioavailability and stability. In this way, monocarbonyl CUR analogs have undergone extensive studies due to their useful biological properties (Zhao, Liu, & Liang, 2013). Several studies focused on the use of (2E, 6E)‐2,6‐bis [(2‐trifluoromethyl) benzylidene] cyclohexanone (so‐called C66), showing that C66 has antioxidant activity (Pan et al., 2012, 2014). Qian et al. (2015), introduced replacement of one trifluoromethyl group with bromine (Justino, Rodrigues, Florencio, & Mira, 2009), to further increase antioxidant activity. Moreover, the same authors in their studies in a mouse model of cardiac injury, have shown that (2E, 6E)‐2‐(2‐bromobenzylidene)‐6‐[(2‐trifluoromethyl) benzylidene] cyclohexanone (called Y20) has increased antioxidant properties in comparison to C66. Based on this, we rationalized that replacing the additional trifluoromethyl group of Y20 with bromine would result in significantly greater antioxidant properties without altering the analog's antiinflammatory properties (Hadzi‐Petrushev et al., 2018).

When dealing with bronchial dysfunction and related complications, the ideal compounds would have both antiinflammatory and antioxidant effects. Recently, CUR’s have been shown to exert antiinflammatory effects by inhibiting the release of proinflammatory cytokines (Camacho‐Barquero et al., 2007; Fu et al., 2014; Sugimoto et al., 2002). Recent studies further demonstrate that CUR attenuates the development of asthma via inhibition of nuclear factor‐κB (NF‐κB) (Oh et al., 2011). In addition, CUR could induce vascular relaxation in porcine coronary artery (Xu, Long, Dai, & Liu, 2007) and goat ruminal artery (Dash & Parija, 2013). Although methanolic extract from *Curcuma longa L* could relax the rat mesenteric artery (Adaramoye et al., 2009), the relaxant effect of CUR on the airway and its mechanism of action are lacking. Also, there have not yet been studies showing the effect of the newly synthesized candidates (2E, 6E)‐2,6‐bis (2‐bromobenzylidene) cyclohexanone (B2BrBC) and C66 on hyperoxia‐induced impairment of ASM relaxation. Therefore, this study was undertaken to test the hypothesis that CUR analogs, B2BrBC and C66 act on the level of TSM in a rat pup model of BPD, and that B2BrBC/C66 supplementation will reverse hyperoxia‐induced increased contraction and impaired relaxation of TSM. Further we sought to determine the antioxidative and antiinflammatory effect of these compounds in our experimental model of BPD.

## 2 MATERIALS AND METHODS

### 2.1 Animals and experimental design

Experiments were performed on Wistar rat pups. On the 5th day of life (P5) pups from two different litters were randomly mixed and assigned to either hyperoxia (*n* = 20; [12 males and 8 females]) or normoxia groups (*n* = 20; [11 males and 9 females]) and exposed for 7 days to hyperoxia >95% O_2_ or kept in room air (normoxia). Animals (mothers) were provided with water and food ad libitum, while a 12‐hr on/12‐hr off light cycle was maintained. Hyperoxic groups were housed with their mothers in a Plexiglas chamber (38 L) and exposed to a continuous flow of O_2_ (2 L/min) for 7 days, while normoxic groups were kept in a commercial rat cage in normal environmental air with their mothers. Mothers were rotated every 24 hr between normoxic and hyperoxic groups to control for hyperoxic exposure. Oxygen concentration within a chamber was monitored continuously via an oxygen analyzer (MiniOX‐1, Ohio Medical Corporation). Pups from both sexes were included in these experiments and no differences were observed between sexes in this study. All experimental procedures were conducted in accordance with the Guiding Principles for Care and Use of Laboratory Animals and were approved by the Ethics Committees’ of our institutions at Ss. Cyril and Methodius University and University of Prishtina.

### 2.2 In vitro measurement of contraction and relaxation of TSM

After the exposure time on day 12 of postnatal life (P12), animals were euthanized by asphyxiation in CO_2_. The trachea was removed and prepared free of serosal connective tissue in an ice‐cold oxygenated Krebs–Henseleit (KH) buffer (concentration in mM: 118.2 NaCl, 25 NaHCO_3_, 4.6 KCl, 1.2 KH_2_PO_4_, 1.2 MgSO_4_, 2.5 CaCl_2,_ and 10% D‐glucose, pH = 7.4; all obtained from Sigma‐Aldrich). Two cylindrical airway segments of 3‐mm length were isolated from the mid‐portions of the tracheas for each animal, and transferred into a tissue‐organ bath containing KH buffer (10 ml) at 37°C, as previously described (Sopi et al., 2012).

Tracheal preparations placed in tissue‐organ baths were suspended between a stainless steel hook at the bottom of the organ‐tissue bath and a force displacement transducer above the bath. TSM tension was measured by the four channel organ bath system integrated with data Acquisition & Control Software (DMT ‐ 750TOBS, Danish Myo Technology) interfaced with computer software (LabChart). The tension of TSM tissue was expressed in grams (g). An initial load ranging from 0.2 to 0.3 g was applied, and then tissues were allowed to equilibrate for 45 min in the organ baths containing KH buffer (10 ml) at 37°C. Preparations were rinsed every 15 min with KH solution during the equilibration time and the solution was continuously aerosolized with a gas mixture (95% O_2_ and 5% CO_2_).

### 2.3 The effect of B2BrBC and C66 on contractile responses of TSM to methacholine

To study the effect of CUR analogs, B2BrBC and C66 on TSM contraction, tracheal preparations from both hyperoxic and normoxic animals were established in the organ baths, then a dose–response curve was constructed using methacholine (MCh, 10^−8^–10^−4^ M) (Sigma‐Aldrich) as an exogenous constrictive agonist in absence or presence of B2BrBC (100 μg/ml) or C66 (100 μg/ml). For in vitro supplementation the newly synthesized CUR analogs were dissolved in distilled water. Tissues were incubated in these compounds for 30 min prior to MCh application. The time between doses of MCh were monitored until the TSM reached plateau contractility. At the end of each cumulative MCh dose–response, the preparations were washed‐out three times every five minutes with warmed KH solution and the TSM were relaxed to baseline for an additional 45 min.

### 2.4 The role of B2BrBC and C66 on relaxant responses of TSM

In order to show the effect of hyperoxia on relaxant responses of TSM, preparations obtained from both hyperoxia‐ and normoxia‐exposed animals were placed in organ baths as described above. After equilibration back to baseline following the methacholine dose–response as described above, a cumulative dose–response curve was then built to find a concentration of the long‐acting muscarinic agonist bethanechol that elicited a 75% of maximal response in TSM. A concentration of 50 μM bethanechol was found to be the optimal dose to elicit 75% of maximal response. Tissues were preconstricted using a single dose of bethanechol (50 μM; Sigma‐Aldrich), then incremental electrical field stimulation (EFS) was applied to the preconstricted TSM through platinum electrodes (5–60 V alternating current [AC] at 50 Hz) for 10 s at 2‐min intervals to induce relaxation, as we have previously described (Sopi et al., 2007, 2008). The relaxation of the TSM was expressed as percentage (%) of preconstricted tension for each preparation.

To determine whether B2BrBC and/or C66 can restore the relaxant responses impaired by hyperoxia, the preparations were incubated in B2BrBC (100 μg/ml) or C66 (100 μg/ml) for 30 min, then EFS was applied.

In another set of experiments, to study the direct relaxant effect of CUR analogs on ASM, cumulative doses of B2BrBC (20, 50, 200 μg/ml) or C66 (20, 50, 200 μg/ml) were applied to preconstricted TSM preparations with bethanechol (50 μM).

### 2.5 The effect of B2BrBC and C66 on antioxidant enzymes

In order to examine the effect of B2BrBC and C66 on the activity of endogenous antioxidant enzymes (CAT, SOD and GPx), under hyperoxia and normoxia conditions pups were treated from P5 to P12 with 20 mg/kg b.w/day of i.p B2BrBC and C66, respectively. B2BrBC and C66 were dissolved in oil. Control animals of hyperoxia and normoxia groups received vehicle only. CAT activity was determined by measuring the degradation of hydrogen peroxide (H_2_O_2_), using the method described by (Claiborne, 1985). SOD activity was determined according to the method described by Marklund and Marklund, (1974), based on the ability of SOD to inhibit the auto‐oxidation of pyrogallol. The activity of GPx was determined according to the method described by (Lawrence & Burk, 1976). The detailed procedure for sample preparation is described in our previous studies (Hadzi‐Petrushev, Jankulovski, Hristov, & Mladenov, 2011; Hadzi‐Petrushev et al., 2012; Mladenov, Gokik, Hadzi‐Petrushev, Gjorgoski, & Jankulovski, 2015). The protein content of the lung samples was determined using the method described by (Lowry, Rosebrough, Farr, & Randall, 1951).

### 2.6 Immuno‐assays for proinflammatory cytokines

Tumor necrosis factor alpha (TNF‐α) and interleukin 1‐beta (IL‐1β), in the lung tissue homogenates were analyzed using the newly developed ELISA for quantitative analysis of TNF‐α and IL‐1β levels from Bender Med‐Systems. The limits of detection of the assays were about 2.3 pg/ml for TNF‐α and 1.0 pg/ml for IL‐1β. Inter‐ and intra‐assay CVs were 8.2% and 9.6% for TNF‐α and 6.5% and 3.2% for IL‐1β. The detailed procedure for sample preparation is described in our previous studies (Hadzi‐Petrushev, Stojkovski, Mitrov, & Mladenov, 2014; Mitrov et al., 2014).

### 2.7 Statistical analysis

Data are expressed as mean ± *SEM*. Statistical significance was determined by two‐way ANOVA with repeated measurements to determine the differences between MCh‐; EFS‐ induced contraction or relaxation responses of hyperoxia versus normoxia; hyperoxia + B2BrBC and/or hyperoxia + C66 versus. hyperoxia control; normoxia + B2BrBC and/or normoxia + C66 versus. normoxia control groups. To compare E_max_ or EC_50_ values between two groups a *t* test was used after checking for normal distribution. To analyze the differences between individual concentrations of particular drugs or individual voltages, post hoc comparison via Tukey–Kramer multiple comparison test was used. The statistical significance of CAT, SOD and GPx activity and TNF‐α and IL‐1β levels was determined by Tukey's post hoc comparisons to identify pairwise differences for all significant ANOVA findings. *p* < .05 was considered as statistically significant.

### 2.8 General characteristics of the chemicals

General characteristics of the chemicals and synthesis of 2,6‐Bis(2‐X‐benzylidene) cyclohexanone derivatives are described in our previous study (Hadzi‐Petrushev et al., 2018).

## 3 RESULTS

### 3.1 Effect of B2BrBC and C66 on hyperoxia‐enhanced TSM contractile responses

**Figure 1:**
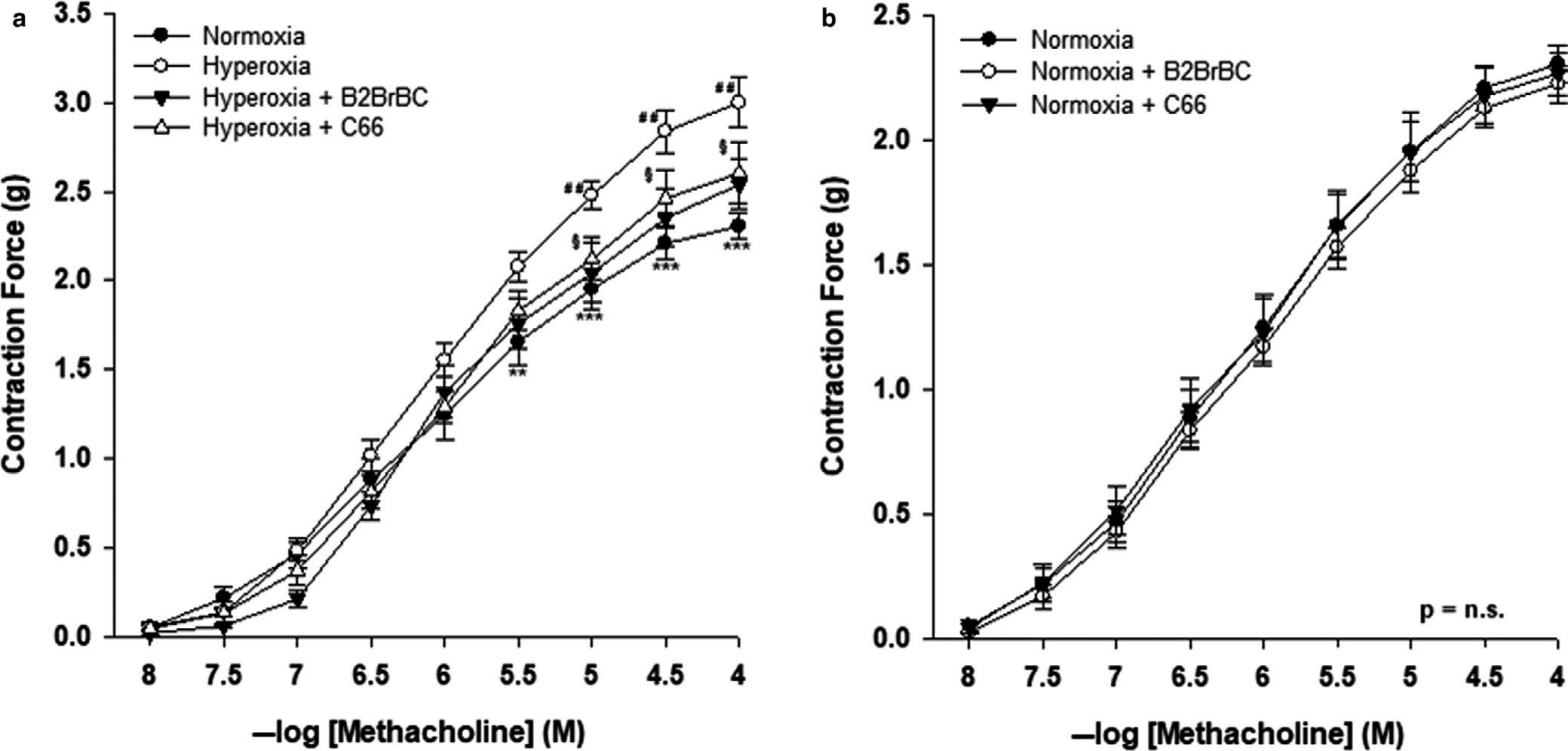
Effect of B2BrBC and C66 on hyperoxia‐induced airway hyperreactivity in rat pups. (a) In hyperoxic group of animals (*n* = 8) contractile responses were greater than in normoxic group (*n* = 8) (*p* < .001). In the presence of B2BrBC (100 μg/ml) or C66 (100 μg/ml) contractile responses of hyperoxic TSM were lower than those recorded in absence of these compounds. (b) In the normoxic group neither B2BrBC nor C66 had any effect on contractile responses of TSM. *Hyperoxia versus Normoxia; ^#^Hyperoxia + B2BrBC versus Hyperoxia control; ^§^Hyperoxia + C66 versus Hyperoxia control. **p < .01; ***p < .001; ^##^ *p* < .01; ^§^ *p* < .05. Data represent mean ± *SEM* of eight experiments for each condition

Hyperoxia significantly increased contractile responses of TSM to MCh compared with contractile responses generated in preparations obtained from normoxic animals (*p* < .001). As shown in Figure 1a, the contractile responses of TSM from pups exposed to hyperoxia (*n* = 8) were significantly greater than those from TSM of normoxic pups (*n* = 8) at concentrations 10^−5.5^–10^−4^ M of MCh. There was a significant increase in E_max_ values of TSM contractile responses in the hyperoxic group compared to the normoxic group (2.94 ± 0.14 g and 2.25 ± 0.07 g, respectively), but the difference in the EC_50_ values [MCh (−log M)] between hyperoxia and normoxia groups was not statistically significant (6.05 ± 0.09 vs. 5.99 ± 0.24, respectively; Table 1).

**Table 1:** Effect of B2BrBC or C66 on ASM hyperreactivity induced by hyperoxia

Group	pEC_50_ (−log M)	E_max_ (g)	*p*‐value (EC_50_)	*p*‐value (E_max_)	
Hyperoxia	6.05 ± 0.09	2.94 ± 0.18	.411	.0003	versus Normoxia
Hyperoxia + B2BrBc	6.18 ± 0.18	2.5 ± 0.14	.264	.025	versus Hyperoxia
Hyperoxia + C66	5.97 ± 0.09	2.5 ± 0.13	.297	.046	versus Hyperoxia
Normoxia	5.99 ± 0.24	2.25 ± 0.07	.267	.562	versus Hyperoxia + B2Br
Normoxia + B2BrBc	6.05 ± 0.06	2.1 ± 0.08	.410	.298	versus Normoxia
Normoxia + C66	6.07 ± 0.12	2.22 ± 0.08	.380	.385	versus Normoxia

In vitro supplementation of tissues with B2BrBC or C66 reversed the hyperoxia‐induced airway hyperreactivity to MCh. In the presence of B2BrBC or C66, TSM contractile responses (Figure 1a; *n* = 8, in both sets of experiments) were significantly decreased at concentrations 10^−5^–10^−4^ M or 10^−4.5^–10^−4^ M of MCh, respectively, as compared to hyperoxia control responses from the same animals in absence of these compounds (overall, *p* < .01 and *p* < .05, respectively). E_max_ values of TSM contractile responses in hyperoxia + B2BrBC and hyperoxia + C66 were 2.52 ± 0.14 g and 2.59 ± 0.13 g, respectively (Table 1). Although, CUR analogs significantly decreased E_max_ values of TSM contractile responses in hyperoxia‐exposed pups, there was no significant difference in EC_50_ values [MCh (−log M)] between hyperoxia control; hyperoxia + B2BrBC and hyperoxia + C66 (6.05 ± 0.09; 6.18 ± 0.18 and 5.97 ± 0.09, respectively; Table 1).

CUR analogs had no significant effect on TSM contractile responses in the normoxic group (Figure 1b).

### 3.2 Effect of B2BrBC and C66 on hyperoxia impaired relaxation of TSM

**Figure 2:**
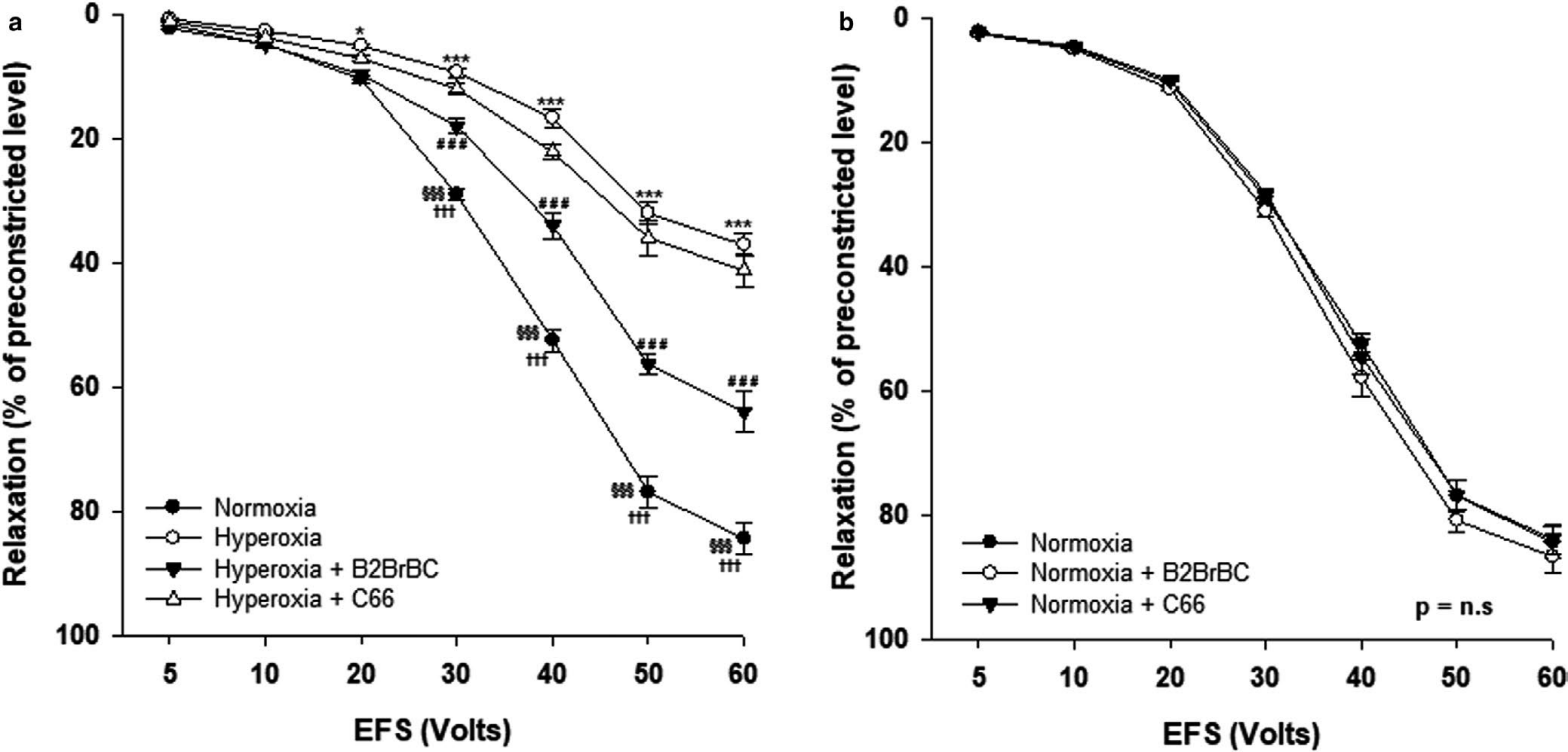
Effect of B2BrBC and C66 on hyperoxia impaired EFS‐induced relaxation of the TSM of rat pups. (a) In hyperoxia‐exposed group of animals (*n* = 6) EFS‐induced relaxation of TSM was reduced as compared to those obtained from normoxia‐exposed group (*n* = 6). In the presence of B2BrBC (100 μg/ml) relaxant responses of hyperoxic TSM were restored and significantly higher than those recorded in absence of this compound (*p* < .001), while the presence of C66 (100 μg/ml) did not have any significant effect on EFS‐induced relaxant responses of TSM. (b) B2BrBC or C66 did not have any effect on EFS‐induced relaxation of TSM in normoxic group. *Hyp eroxia versus Normoxia; ^#^Hyperoxia + B2BrBC versus Hyperoxia control; ^§^Normoxia control versus Hyperoxia + B2BrBC; ^†^Normoxia control versus Hyperoxia + C66. *p < .05; ***p < .001; ^###^ *p* < .001; ^§§§^ *p* < .001; ^†††^ *p* < .001. Data represent mean ± *SEM* of six experiments for each condition

In hyperoxic animals, relaxation of TSM induced by EFS overall was significantly reduced, as compared to room air exposed animals (*p* < .001). As shown in Figure 2a, the relaxant responses of TSM from pups exposed to hyperoxia (*n* = 6) were significantly attenuated compared to TSM of room air pups (*n* = 6), from 20 to 60 V. The data of relaxant responses in hyperoxic and normoxic group ranged from 0.81 ± 0.20% at 5 V to 37.40 ± 1.88% at 60 V, and from 2.39 ± 0.26% at 5 V to 84.37 ± 2.61% at 60 V, respectively.

B2BrBC reversed the effects of hyperoxia attenuation of relaxant responses in TSM, but C66 did not show any significant effect on relaxant responses under these conditions. As shown in Figure 2a, the relaxant responses of TSM to EFS in the hyperoxic group were significantly increased (overall, *p* < .001) when the preparations were preincubated in B2BrBC as compared to the relaxant responses in absence of this compound, particularly at higher voltages (30–60 V). The relaxation data under condition of supplementation with B2BrBC ranged from 1.66 ± 0.51% at 5 V to 63.98 ± 3.38% at 60 V. Relaxant responses of TSM to EFS in the hyperoxic group did not significantly differ in the presence of C66. The data ranged from 1.15 ± 0.27% at 5 V to 41.27 ± 2.81% at 60 V. Both, B2BrBC or C66 did not show any significant effect on EFS‐induced TSM relaxation in the normoxic group (Figure 2b).

### 3.3 B2BrBC and C66 induced concentration‐dependent relaxation of TSM

**Figure 3:**
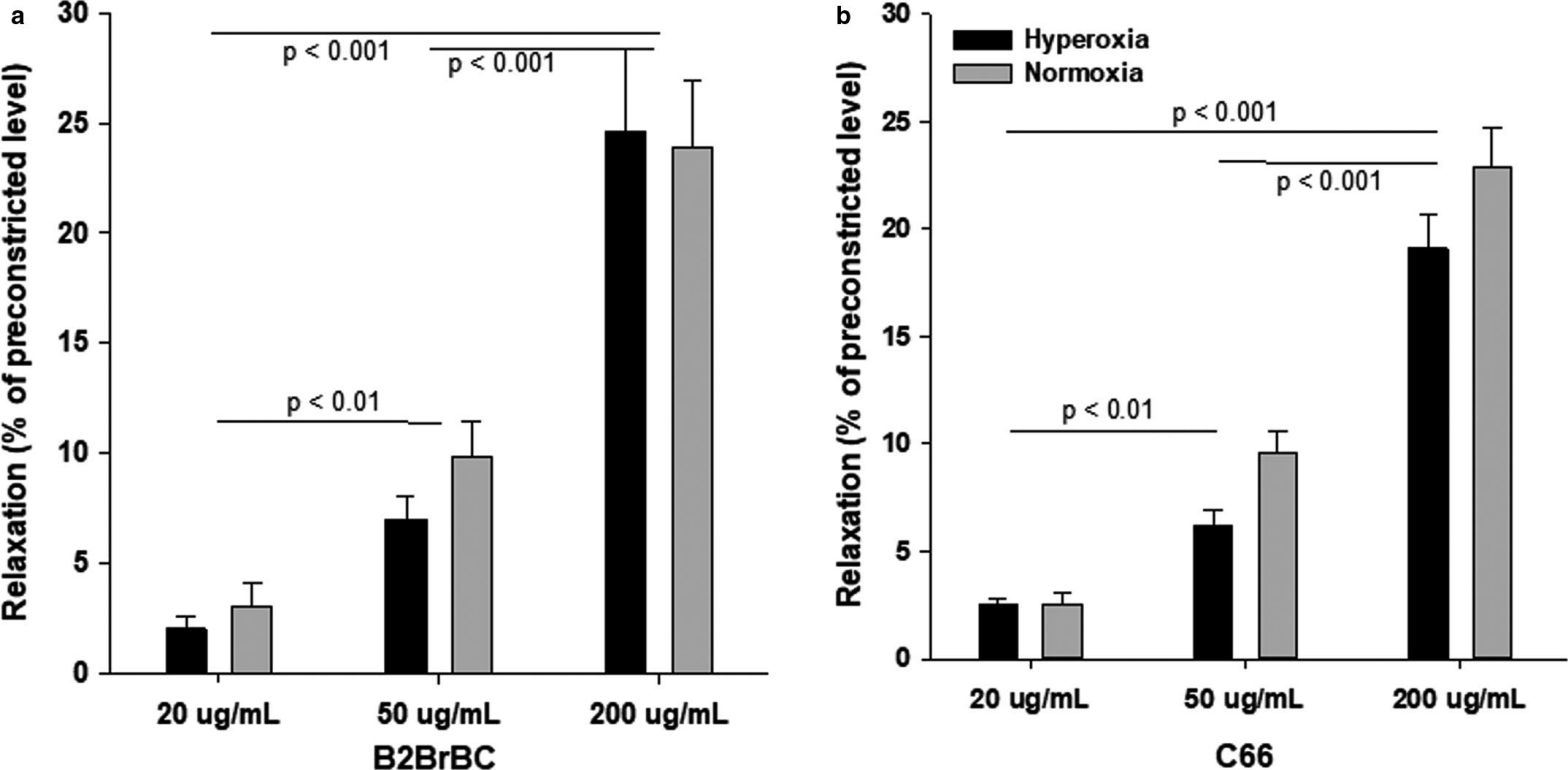
Relaxant responses of airway smooth muscle towards different concentrations of B2BrBC‐(A) and C66‐(B). (a,b) B2BrBC and C66, both induced direct concentration‐dependent relaxation of preconstricted TSM, and the difference was significant between different doses within a same group and across different groups (Hyperoxia & Normoxia – 200 μg/ml versus Hyperoxia & Normoxia – 50 or 20 μg/ml, *p* < .001; Hyperoxia & Normoxia – 50 μg/ml versus Hyperoxia & Normoxia – 20 μg/ml, *p* < .01). Data represent mean ± *SEM* of eight experiments

In order to define the direct relaxant effect of CUR analogs on TSM, preconstricted TSM were challenged with different concentrations of B2BrBC or C66 (20, 50 and 200 μg/ml). B2BrBC induced a concentration‐dependent relaxation of TSM in both, hyperoxic (*n* = 8) and normoxic (*n* = 6) groups (Figure 3a). There was significant relaxation with increasing concentrations of B2BrBC within each group (*p < *.001), but not a significant difference between normoxic and hyperoxic groups at the same dose.

Interestingly, the presence of C66 did not restore EFS‐induced relaxation of TSM obtained from hyperoxic rat pups (Figure 2a), but in preconstricted TSM C66 triggered concentration‐dependent relaxation in both hyperoxic (*n* = 8) and normoxic (*n* = 6) groups (Figure 3a,b). There was a significant difference in relaxant effects observed with increasing concentrations of C66 within each group (*p < *.001), but not a significant difference between normoxic and hyperoxic groups at the same dose.

### 3.4 Effect of B2BrBC and C66 on antioxidant activity

**Figure 4:**
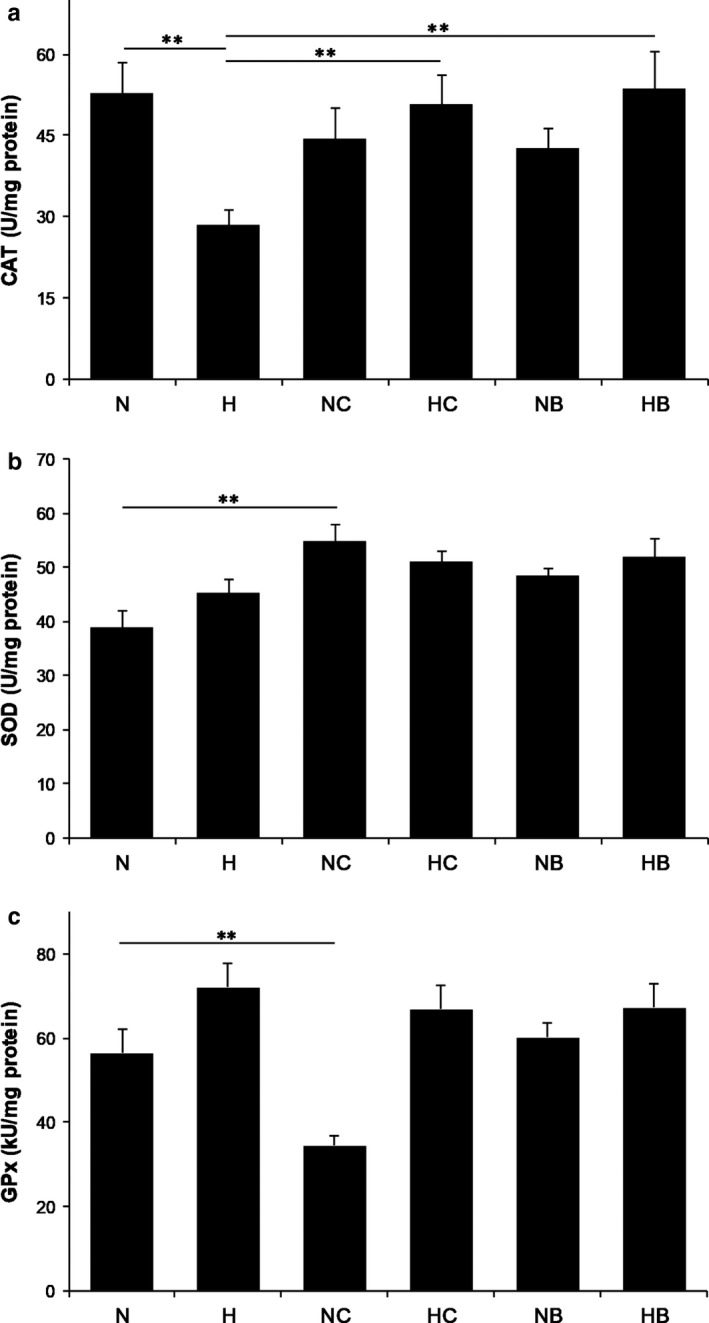
Effect of B2BrBC and C66 on CAT‐(A), SOD‐(B) and GPx‐(C) activity. (a) Hyperoxia induced significant decrease in catalytic activity of CAT (*p* < .01). B2BrBC and C66 significantly preserved catalytic activity of CAT during hyperoxic exposure (*n* = 8; *p* < .01). (b,c) SOD and GPx showed nonsignificant increase in their activity under hyperoxic conditions (*n* = 8; *p* = .059 and *p* = .053, respectively). B2BrBC and C66 did not cause any change in the activity of CAT and SOD in normoxic pups. GPx was significantly decreased in the normoxic pups treated with C66 (*n* = 8; *p* < .01), while B2BrBC did not show any effect (*n* = 8; *p* > .05). *p < .05; **p < .01; n, number of pups; N, normoxic; H, hyperoxic; NC, normoxic treated with C66; HC, hyperoxic treated with C66; NB, normoxic treated with B2BrBC; HB, hyperoxic treated with B2BrBC

In comparison to the normoxic group, among the studied enzymes only CAT had significantly decreased catalytic activity in the hyperoxia‐exposed group (*n* = 8; *p < *.01), (Figure 4a). In terms of treatment, both CUR analogs possess high antioxidant capacity, expressed through preservation of catalytic activity of CAT during hyperoxic exposure (*n* = 8; *p* < .01). The treatment with CUR analogs did not show a significant change in the activity of CAT in normoxic animals. There was a tendency of increased lung SOD and GPx activity in hyperoxia‐exposed pups (Figure 4b,c; *p* = .059 and *p* = .053, respectively). Interestingly, in the normoxic controls treated with C66, SOD was significantly increased (*n* = 8; *p* < .01) and GPx was significantly decreased (*n* = 8; *p* < .01), while B2BrBC did not show an effect in normoxic controls.

### 3.5 Effect of B2BrBC and C66 on proinflammatory cytokines

**Figure 5:**
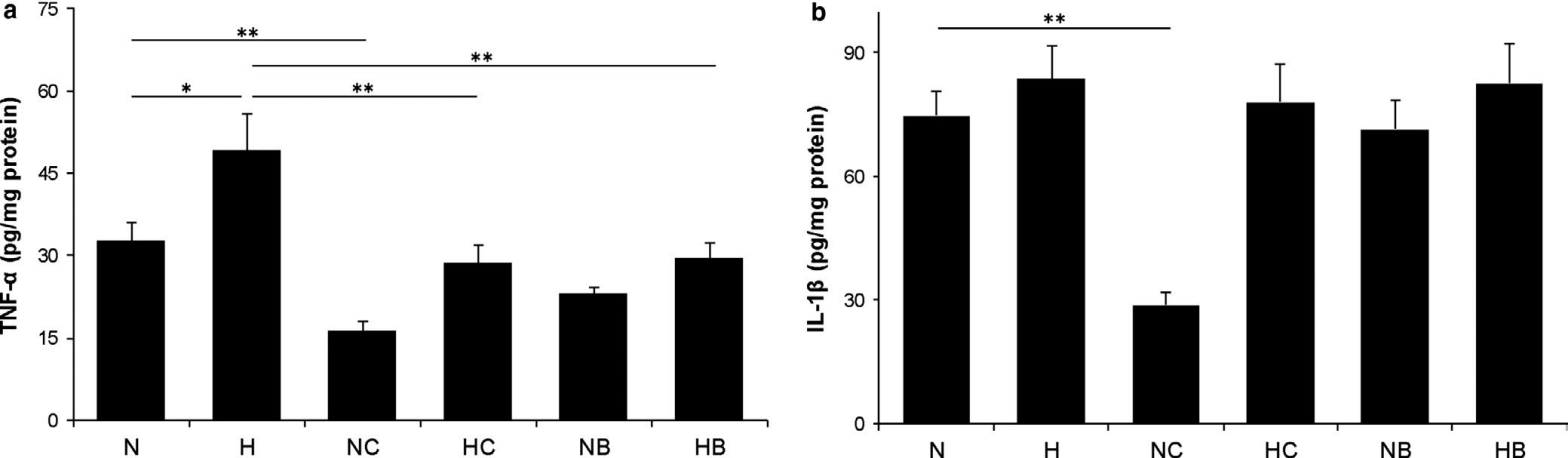
Effect of B2BrBC and C66 on TNF‐α‐(A) and IL‐1β‐(B). (a) Hyperoxia caused a highly significant increase on TNF‐α (*p* < .01). B2BrBC and C66 caused significant prevention of hyperoxia‐induced TNF‐α increase (*n* = 8; *p* < .05), while in normoxic pups only C66 caused significant decrease in TNF‐α (*n* = 8; *p* < .05). (b) In relation to IL‐1β, significant changes were found only in C66‐treated normoxic pups (*n* = 8; *p* < .05). *p < .05; **p < .01; n, number of pups; N, normoxic; H, hyperoxic; NC, normoxic treated with C66; HC, hyperoxic treated with C66; NB, normoxic treated with B2BrBC; HB, hyperoxic treated with B2BrBC

In relation to proinflammatory cytokines (TNF‐α and IL‐1β), this study showed significantly increased expression of TNF‐α in conditions of hyperoxia compared to normoxia (*n* = 8; *p* < .05). Treatment with both CUR analogs resulted in decreased expression of TNF‐α in both, normoxia and hyperoxia (*n* = 8; *p* < .01; *p* < .01, respectively) (Figure 5a). IL‐1β did not significantly differ in hyperoxia‐exposed pups, but C66‐treated normoxic pups had significantly decreased expression compared to normoxic controls (*n* = 8; *p* < .01) (Figure 5b).

## 4 DISCUSSION

Our previous studies in neonatal rats have shown that long‐term hyperoxic exposure disrupts the tracheal relaxation mechanisms (Ali et al., 2011; Sopi et al., 2007) and increases contraction of intrapulmonary airways in neonatal rats (Sopi et al., 2008) In this study, we have shown that exposure to hyperoxia causes a decrease in EFS‐induced relaxation of TSM. We addressed the question whether redox modulation in the hyperoxic rat pup's lungs can be attenuated with postexposure CUR treatment (Rao, 1997). As expected, TSM preparations from hyperoxia‐exposed rat pups, showed higher reactivity toward moderate and higher doses of methacholine, compared to those from normoxic rat pups. This finding is based on several mechanisms, which include an imbalance of increased contractile mediators accompanied with decreased relaxant mediators during hyperoxia (Belik et al., 2003).

Supplementation of tracheal preparations with CUR analogs, B2BrBC or C66, significantly reduced contractile responses to MCh in hyperoxic animals. Pretreatment with CUR analogs did not cause any change in the EC_50_ but significantly decreased the E_max_ contractile responses in TSM derived from hyperoxic animals; while no effect was observed in normoxic control animals. A lack of differences in EC_50_ values of hyperoxia‐exposed TSM preparations pretreated with B2BrBC or C66 compared to untreated, suggests stabilized constrictive mediators’ release from the TSM. We speculate its physiological role is based on the induction of mechanisms participating in the processes concealed by molecules generated during oxidative processes such as H_2_O_2_.

Besides, the preventive effect on hyperoxic airway hyperreactivity, B2BrBC restored the impaired relaxant responses induced by EFS. In addition to the restoration of impaired relaxant responses, both, CUR analogs induced direct relaxation of TSM in a dose‐dependent manner in preconstricted TSM. Our findings are in consent with published results showing the relaxant effect of curcumin on the smooth muscle of different organs. Curcumin and its derivatives relax the rat aorta through a NO‐independent pathway (Sasaki et al., 2003). A direct relaxant effect of curcumin was shown in preconstricted guinea pigs’ gallbladder smooth muscle, through multiple signaling pathways, including protein kinase C, Ca^2+^ influx and K^+^ channels (Kline & Karpinski, 2015). Closer scrutiny of relaxation data (Figure 3a,b) show that CUR analogs induce relaxation of TSM in a dose‐dependent manner. We are of the opinion that both B2BrBC and C66 modulate the level of released H_2_O_2_ from the hyperoxic airways in amounts that are below the transitory concentration necessary to induce contraction of smooth muscle (Shi et al., 2007).

Hyperoxia causes the intrinsic imbalance between pro‐ and antioxidant mechanisms leading to oxidative stress and production of specific oxidative molecules on the level of the airways, such as H_2_O_2_ (Audi et al., 2018; Gil‐Ortega et al., 2014). Hyperoxia decreased CAT activity, an enzyme that scavenges H_2_O_2_. H_2_O_2_ interacts with other molecules and triggers signaling pathways that lead to contraction of smooth muscle (Erdei et al., 2007). Our assumption is that released H_2_O_2_ in the TSM promotes constriction of TSM as a result of diminished CAT activity in the lungs of rat pups exposed to hyperoxia which was preserved by CUR analogs. This restoration of CAT activity parallels the hyperreactive and attenuated relaxative physiological responses of TSM affected by hyperoxic exposure, which were effectively reversed by treatment of preparations with CUR analogs. CAT causes conversion of H_2_O_2_ into water and O_2_, and thus eliminates the paracrine effects of H_2_O_2_. Decreased CAT activity in conditions of hyperoxia might be the direct result of exhausted CAT catalytic activity, due to the persistent production of H_2_O_2_. Our results show a tendency of increased SOD levels in hyperoxic animals, however it was not significant. In relation to CUR analogs, the activity of SOD increased only in the normoxic group treated with C66. Thus, C66 as an enhancer of SOD activity could be effective in the improvement of hyperoxia‐induced impairment. We also assume that both CUR analogs have the capacity to attenuate H_2_O_2_‐mediated mitochondrial dysfunction by both, increasing the expression levels of antioxidant enzymes (Selvam, Subramanian, Gayathri, & Angayarkanni, 1995) and acting as direct ROS scavengers (Kimura, 2014).

Further, based on the obtained results it is more than evident that both B2BrBC and C66 possess antiinflammatory properties based on their ability to prevent production of proinflammatory cytokines during hyperoxia. There are several mechanisms described for CUR derivatives to exercise their antiinflammatory activity. Liu et al. (2015), reported that derivatives of CUR might alleviate airway inflammation in asthma through the Nrf2/HO‐1 pathway. Other studies suggest that intranasal CUR (2.5 and 5.0 mg/kg) regulates airway inflammation and airway obstruction by modulating cytokine levels (IL‐4, IL‐5, IFN‐α, and TNF‐α) and sPLA2 activity thereby inhibiting PGD_2_ release and COX‐2 expression (Kumari, Dash, & Singh, 2017; Masella, Di Benedetto, Varì, Filesi, & Giovannini, 2005; Subhashini, Chauhan, Dash, Paul, & Singh, 2016). The same studies also reveal that suppression of p38 MAPK, ERK 42/44 and JNK 54/56 activation, with CURs protects against asthma progression (Kumari et al., 2017; Masella et al., 2005; Subhashini et al., 2016). This supports our findings that C66 has more pronounced anti‐inflammatory effects in comparison to B2BrBC, as measured by the proinflammatory cytokine IL‐1β in normoxic counterparts. We also believe that hyperoxia by itself induces stronger inflammatory reaction that overcomes antiinflammatory capacity of C66. In addition, taking that hyperoxia induces production of different sets of cytokines from one, and cross‐reactivity of these with the other endogenously produced mediators from the other side, open further speculation concerning the complexity of the obtained results.

It seems that the reactivity of B2BrBC is dependent on the atomic charges of the olefinic carbon atoms (Hadzi‐Petrushev et al., 2018), and the torsion angles between the aryl ring and the adjacent unsaturated groups. The reactivity of this system can be tuned by the introduction of various electron withdrawing groups in the phenyl rings of which the ortho position (X = NO_2_, CF_3_ or halides) seemed most promising. Based on this, B2BrBC could be classified as a stronger antioxidant and weaker “anti‐inflammatory” in comparison to C66. Actually, B2BrBC is the α, β‐unsaturated monocarbonyl CUR analog known as a good Michael acceptor which classifies it as a strong antioxidant (Hadzi‐Petrushev et al., 2018). This stronger antioxidative capacity is also observed through increased relaxation in hyperoxic preparation pretreated with B2BrBC.

The model used in this study successfully characterizes the protective role of B2BrBC and C66 against hyperoxia‐induced hyperreactivity and impairment of relaxation in TSM. However, we are aware that the study has some limitations, as physiological experiments are performed in isolated preparations which are disconnected from the systemic circulation and central nervous system, which are important for in vivo airway physiology. Pups were exposed to neonatal hyperoxia starting at age P5 which represent the transition day from saccular to alveolar phase of lung development in rats (O’Reilly & Thebaud, 2014). The 5th day of life, as starting point for exposure to oxygen might not be critical to induce strong structural changes in the airways and interstitial parenchyma of lungs to represent remodeling, but it is critical day to induce functional changes in respiratory system, particularly to trigger airway hyperreactivity that was proven in our previous publications (Sopi et al., 2007, 2012). Lungs at this age are immature, which is one of the risk factors of BPD (Speer, 2006).

This study also lacks the dose–response effect of the newly synthesized CUR analogs on contractile responses, because we aimed to reverse contractile responses to normal level but not to completely diminish, and the single dose we used was appropriate to restore the balance between contractile and relaxant processes in airway smooth muscle cells.

In conclusion, our results show the effects of B2BrBC and C66 to counteract neonatal hyperoxic hyperreactivity on TSM and that H_2_O_2_ is likely an important hyperoxia‐derived constrictive mediator in TSM. B2BrBC and C66 preserve the activity of CAT, critical for regulation of H_2_O_2_ production in hyperoxic conditions. The last mechanism may be crucial in the amelioration of oxidative dis‐balance during hyperoxia. Moreover, CUR analogs restored redox balance in the lung and reversed impaired relaxation of TSM in hyperoxic conditions. While this is the first study using these newly synthesized CUR analogs in neonatal hyperoxia, future studies are planned to investigate additional protective effects of these and other newly synthetized CUR analogs in various in vitro and in vivo preparations. CUR analogs may be promising new therapies for neonatal hyperoxic airway and lung disease, such as BPD.

## CONFLICT OF INTEREST

The authors declare that they have no conflict of interest.

## AUTHOR CONTRIBUTIONS

RBS, MM and RS conceived and designed the study, supervised the experiments and data analysis, interpreted the results, and contributed to the manuscript; MS and QT performed a major part of the experiments and wrote the manuscript; SR and IK performed in vitro experiments; RS, HG, and AK participated in designing the study and contributed to finalizing the manuscript; NHP, VM, JB, and MA performed the in vivo experiments. All authors read and approved the final draft of the manuscript.

## Ethics approval

This research protocol was approved by the Institutional Review Boards of University St. Cyril and Methodius in Skopje (No.009–2018) and University of Prishtina.
