# Corrigendum: Curcumin analogs (B2BrBC and C66) supplementation attenuates airway hyperreactivity and promote airway relaxation in neonatal rats exposed to hyperoxia

**DOI:** 10.14814/phy2.14667

**Published:** 2021-01-03

The authors of the article by Stamenkovska et al. (2020) noticed an error in the affiliation for several of the co‐authors, in that both Kosovo, Serbia were included. The only country listed for this affiliation should have been Kosovo. Here is the corrected affiliation:

2 Department of Premedical Courses‐Biology, Faculty of Medicine, University of Prishtina, St. Martyrs' Boulevard n.n., Prishtina, Kosovo

The authors apologize for this mistake.
